# Factors associated with the etiological uncertainty of drug reactions: a cross-sectional study in southern Brazil^☆^^☆☆^

**DOI:** 10.1016/j.abd.2021.04.002

**Published:** 2021-05-27

**Authors:** Fabiana Bazanella de Oliveira, Paula Chiamenti, Roberta de Freitas Horn, Renan Rangel Bonamigo

**Affiliations:** a Universidade Federal de Ciências da Saúde, Porto Alegre, RS, Brazil; b Faculty of Medicine, Universidade Federal do Rio Grande do Sul, Porto Alegre, RS, Brazil; c Hospital de Clínicas de Porto Alegre, Porto Alegre, RS, Brazil

Dear Editor,

We report a retrospective and cross-sectional study, conducted through the analysis of medical records of hospitalized patients evaluated by the Dermatology Service of Hospital de Clínicas de Porto Alegre, with the clinical or histopathological diagnosis of drug reaction, from January 2011 to January 2019. All drug reaction patterns were included, regardless of whether the causative drug was identified or not. Cases in which the diagnosis of drug reaction (DR) was not fully established (when the condition could also correspond to infectious, autoimmune or non-drug-related reactions) were excluded. The DR that were considered severe were the following: Stevens-Johnson Syndrome (SJS), overlap SJS /Toxic Epidermal Necrolysis (TEN), TEN, Drug-Hypersensitivity Syndrome such as DRESS, Acute generalized exanthematous pustulosis (AGEP), and Erythroderma.^1^

The frequencies of each demographic and clinical variable were described. The statistical analysis was performed using the SPSS v18.0 program. Nonparametric tests were used to assess continuous variables, as the data distribution was non-linear, according to the Kolmogorov-Smirnov test. The Mann-Whitney U test was used for these variables. For categorical variables, associations were assessed using the Chi-squared test. Multiple logistic regression was performed to assess factors that could or not be associated with a higher probability of etiological diagnosis. A result with p < 0.05 was considered significant.

The study identified 354 patients with drug reactions, with an average of 44.25 new cases per year, an annual incidence of 1.4 cases per 1,000 general admissions. The mean age was 46.08 (± 21.307) and 209 (59%) were women. Most had some type of comorbidity (88.7%; n = 314) (Table 1). There was a higher number of non-severe drug reactions (72.3%; n = 256), with maculopapular exanthema being the most common form (58.9%; n = 212). Most patients did not have systemic findings (72.3%; n = 256) (Table 1).

**Table 1:** Characteristics of drug reaction patients - Hospital de Clínicas de Porto Alegre, 2011–2019.

Mean Age (± SD)		46.08 (± 21.307)
**Sex, %(n)**		
	Female	59 (209)
	Male	41 (145)
Number of medications in use, median (IQR)		7 (I 0–27)
Number of suspected drugs, median (IQR)		3 (1–16)
Medication causing drug reaction identified, % (n)		33.3 (117)
**Severe drug reaction, % (n)**		27.6 (98)
	Overlap SJS/NET	8.5 (30)
	Erythema multiforme	6.5 (23)
	DRESS	11 (39)
	AGEP	0.6 (2)
	Erythroderma	1.1(4)
**Mild drug reaction,% (n)**		72.3 (256)
	Exanthema	59.9 (212)
	Fixed pigmented erythema	4.5 (16)
	Photosensitivity	3.4(12)
	Lichenoid Eruption	0.3 (1)
	SDRIFE	0.8 (3)
	Dysesthesia of hands and feet	0.3 (1)
	Acneiform reaction	0.8 (3)
	Small vessel cutaneous vasculitis	1.1 (4)
	Urticaria	1.1 (4)
**Extra-cutaneous involvement, % (n)**		27.7 (98)
	Eosinophilia	18.1 (64)
	Liver function alteration	13.3 (47)
	Fever	11.6 (41)
	Renal function alteration	4 (14)
	Pneumonitis	0.3 (1)
	Lymphadenomegaly	1.1 (4)
	Pancytopenia	0.3 (1)
**Presence of comorbidity, % (n)**		88.7 (314)
	SAH	33.1 (117)
	Diabetes mellitus	14.4 (51)
	Ischemic heart disease	5.6 (20)
	COPD	4.2 (15)
	Asthma	1.7 (6)
	Neoplasia	23.7 (84)
	HIV	11 (39)
	Chronic Kidney Disease	7.1 (25)
	Epilepsy	4.8 (17)
	Psychiatric Disease	6.5 (23)
	Heart failure	4.8 (17)
	Cirhosis	2.5 (9)
	Stroke	5.6 (20)

SD, Standard Deviation; IQR, Interquartile Range; %, Percentage, n, Number.

Most cases of drug reactions occurred during the use of more than one medication (79.7%; n = 282). The medians of the number of drugs used at the time of the skin rash and of the suspected drugs were seven (IIQ 0–27) and three (IIQ 1–16), respectively.

In 66.9% (n = 237) of the cases, it was not possible to identify the causative drug. Carbamazepine was the drug most commonly related to the definitive diagnosis (n = 18; 15.4%); followed by phenytoin (n = 16; 13.7%) and dipyrone (n = 11; 9.4%). The main groups were aromatic anticonvulsants (31.6%; n = 37), followed by antimicrobials (29%; n = 34). Of the antimicrobials, penicillins were the main drug class (n = 13) (Table 2).

**Table 2:** Groups of medications identified as causing drug reactions.

Groups of medications	% (n)
Aromatic Anticonvulsants	31,6 (37)
Antimicrobials	29 (34)
Penicillins	11,1 (13)
Sulfonamides	8,5 (10)
Cephalosporins	4,2 (5)
Vancomycin	2,5 (3)
Fluoroquinolones	1,7 (2)
Macrodantin	0,8 (1)
Hydroxychloroquine	1,7 (2)
Allopurinol	1,7 (2)
Clopidogrel	0,8 (1)
Contrast	0,8 (1)
Fluconazole	0,8 (1)
Gemfibrozil	0,8 (1)
Homeopathic drugs	0,8 (1)
Valproic acid	0,8 (1)
Total	100 (117)

Aromatic anticonvulsants: Carbamazepine, Phenytoin, Phenobarbital, Lamotrigine.

Non-steroidal anti-inflammatory drugs: Ibuprofen, Nimesulide, Diclofenac.

Antiretrovirals: Ritonavir, Etretinate.

Corticoid: Prednisone.

Penicillins: Amoxicillin-Clavulanate, Amoxicillin, Ampicillin, Ampicillin-Sulbactam, Oxacillin; Sulfonamides: Sulfamethoxazole-trimethoprim, Sulfadiazine; Cephalosporins: Cefepime, Cefuroxime; Fluoroquinolones: Levofloxacin, Ciprofloxacin.

There was no statistical difference in relation to age (p = 0.429), sex (p = 0.633), and presence of comorbidities (p = 0.321) between cases with or without a defined etiology (Table 3). Patients whose causative drug was not identified used a higher number of medications (median of eight, IQR 0–27) and a higher number of suspected drugs (median of three, IQR 1–16), compared to patients who had an etiological definition (medians of four, IQR 0–26 and one, IQR 1–7, respectively), p < 0.000 (Table 3). Patients with severe drug reactions had a higher proportion of identification of the causative drugs than the ones with non-severe drug reactions (p = 0.007). We can observe a general comparison between patients with and without a defined etiology in Table 3.

**Table 3:** Profile of patients with etiologically defined and undefined drug reactions.

	Drug reactions with defined etiology	Drug reactions with undefined etiology	p
**Age (± SD)**	40.49 (±22.069)	46.86 (±20.924)	p = 0.429^b^
**Sex, %(n)**			
Female	32.1 (67)	67.9 (142)	p = 0.633^c^
Male	34.5 (50)	65.5 (95)	
Number of medications in use at the time of the skin rash, median (IQR)	4 (0–26)	8 (0–27)	p < 0.000^b^
Number of suspected medications, median (IQR)	1 (1–7)	3 (1–16)	p < 0.000^b^
Severe drug reaction^a^	36.8 (43)	23.2 (55)	p = 0.007^c^
Mild drug reaction	63.2 (74)	76.8 (182)	p = 0.007^c^
**Comorbidities**			
Yes	32.2 (101)	67.8 (213)	p = 0.321^c^
No	40 (16)	60 (24)	

a Severe pharmacodermia: DRESS, Overlap SJS/NET, AGEP, Erythroderma, Erythema Multiforme.

b Mann-Whitney *U* test.

c Chi-squared.

The factors that could be related to a greater probability of achieving the etiological diagnosis were evaluated. The following factors showed statistical significance for attaining an etiological definition for the drug reactions: lower number of drugs in use (p < 0.0001), lower number of suspected drugs (p < 0.0001), and severe drug reactions (p = 0.007). The first two factors are characteristic of patients receiving multiple drugs.

Using logistic regression analysis, and after the adequate adjustments, the lowest number of suspected drugs (OR = 0.29, 95% CI 0.22–0.38) was found to be definitely important for the etiological definition of drug reactions. It is noteworthy that in this stage of the evaluation, the total number of drugs in use was not included, since factors that are closely related to each other or that use similar parameters should not be included in the multivariate analysis (Table 3).

It was already suspected that the use of multiple drugs is an important contributing factor for the impossibility of an etiological diagnosis in drug reactions, which is corroborated by our results.^2^, ^3^, ^4^

It is known that multiple drug use is one of the main obstacles in defining the causative drug. In our sample, 79.7% of patients used more than one drug at the time of the skin rash and, consequently, had more than one suspected drug. This showed to be similar to the study by Beniwal et al. which demonstrated that 74% of patients were using multiple drugs at the time of the drug reaction.^2^

Most of the evaluated patients did not have the causative drug identified, corresponding to 66.9% (n = 237) of the cases. This was related to the high percentage of multiple drug use, 79.7% (n = 282) and a higher median of drugs in use than those described in other studies. The median number of drugs used by the patients in our study was seven (IQR 0–27) and the mean number of suspected drugs was three (IQR 1–16). These numbers are higher than those of other studies, such as the one by Beniwal et al., which was 2.09 (IIQ 1–6) and that of Thakkar et al., which observed a mean number of 1.52 (± 0.80).^2^, ^3^

There are few studies in the literature describing cases in which an etiological diagnosis was not established, since the absence of a diagnosis of the precipitating drug is an exclusion criterion in most protocols. In the case of the study by Zhao J et al.,^4^ the causative drug was not established in only 13% of patients, who were using more than one medication. However, cases that did not have defined causal drugs or did not have suspected drugs, and if patients were using more than five drugs, were excluded at the selection phase.^4^ This is in contrast with our study, considering that the median number of drugs in use was higher than five.

In conclusion, in a tertiary referral hospital in southern Brazil, drug reactions are frequent, and the possibility of not having a defined etiology is high. Multiple drugs use is the main factor associated with the etiological ambiguity of drug reactions.

## Financial support

FUNADERM - Fundo de Apoio à Dermatologia, Sociedade Brasileira de Dermatologia, Rio de Janeiro, RJ, Brazil.

## Authors’ contributions

Fabiana Bazanella de Oliveira: Statistical analysis; approval of the final version of the manuscript; design and planning of the study; drafting and editing of the manuscript; collection, analysis, and interpretation of data; effective participation in research orientation; intellectual participation in the propaedeutic and/or therapeutic conduct of the studied cases; critical review of the literature; critical review of the manuscript.

Paula Chiamenti: Collection, analysis, and interpretation of data.

Roberta de Freitas Horn: Collection, analysis, and interpretation of data.

Renan Rangel Bonamigo: Approval of the final version of the manuscript; design and planning of the study; effective participation in research orientation; intellectual participation in the propaedeutic and/or therapeutic conduct of studied cases; critical review of the manuscript.

## Conflicts of interest

None declared.
